# Ternary Nanocomposite System Composing of Graphene Nanoplatelet, Cellulose Nanofiber and Jatropha Oil Based Waterborne Polyurethane: Characterizations, Mechanical, Thermal Properties and Conductivity

**DOI:** 10.3390/polym13213740

**Published:** 2021-10-29

**Authors:** Mohamad Ridzuan Amri, Faizah Md Yasin, Luqman Chuah Abdullah, Syeed Saifulazry Osman Al-Edrus, Siti Fatahiyah Mohamad

**Affiliations:** 1 Institute of Tropical Forestry and Forest Product, Universiti Putra Malaysia, Serdang 43400, Selangor, Malaysia; mridzuan.work@gmail.com; 2 Department of Chemical and Environmental Engineering, Faculty of Engineering, Universiti Putra Malaysia, Serdang 43400, Selangor, Malaysia; 3 Institute of Advance Technology, Universiti Putra Malaysia, Serdang 43400, Selangor, Malaysia; 4 Radiation Processing and Technology Division, Malaysia Nuclear Agency, Bangi 43000, Selangor, Malaysia; fatahiyah@nuclearmalaysia.gov.my

**Keywords:** nanocellulose, graphene nanoplatelet, bio-based polyol, biopolymer, bio-based film, bio-composite

## Abstract

This work aims to evaluate the performance of graphene nanoplatelet (GNP) as conductive filler with the presence of 0.5 wt.% cellulose nanofiber (CNF) on the physical, mechanical, conductivity and thermal properties of jatropha oil based waterborne polyurethane. Polyurethane was made from crude jatropha oil using an epoxidation and ring-opening process. 0.5, 1.0, 1.5, 2.0 wt.% GNP and 0.5 wt.% CNF were incorporated using casting method to enhance film performance. Mechanical properties were studied following standard method as stated in ASTM D638-03 Type V. Thermal stability of the nanocomposite system was studied using thermal gravimetric analysis (TGA). Filler interaction and chemical crosslinking was monitored using Fourier-transform infrared spectroscopy (FTIR) and film morphology were observed with field emission scanning electron microscopy (FESEM). Water uptake analysis, water contact angle and conductivity tests are also carried out. The results showed that when the GNP was incorporated at fixed CNF content, it was found to enhance the nanocomposite film, its mechanical, thermal and water behavior properties as supported by morphology and water uptake. Nanocomposite film with 0.5 wt.% GNP shows the highest improvement in term of tensile strength, Young’s modulus, thermal degradation and water behavior. As the GNP loading increases, water uptake of the nanocomposite film was found relatively small (<1%). Contact angle test also indicates that the film is hydrophobic with addition of GNP. The conductivity properties of the nanocomposite film were not enhanced due to electrostatic repulsion force between GNP sheet and hard segment of WBPU. Overall, with addition of GNP, mechanical and thermal properties was greatly enhanced. However, conductivity value was not enhanced as expected due to electrostatic repulsion force. Therefore, ternary nanocomposite system is a suitable candidate for coating application.

## 1. Introduction

Jatropha oil is a promising candidate for polyurethane intermediate. *Jatropha curcas* is a small tree and mostly planted in tropical and sub-tropical region. Jatropha oil consist of high concentration of toxic ingredient of phorbol esters, which made jatropha oil as non-edible oil. Due to this, jatropha oil price is unaffected by fast development in food industry, thus make it an interesting candidate among vegetable oils. Jatropha oil consist of substantial amount of unsaturated fatty acid. Those unsaturated fatty acid include oleic acid (18:1), linoleic acid (18:2), palmitoleic acid (16:1) and linoleic acid (18:3) [1]. Overall, the iodine value of jatropha oil in in average of 105 g I_2_/100g. Iodine value shows amount of unsaturated fatty acid content in chemical solution.

Studies show that most conductive polymer composites have a range of excellent properties, which are high thermal stability, corrosion resistance, great conductivity and high strength, and modulus properties [1,2,3,4,5,6,7]. This properties enhancement is influenced by two factors which are good choice of polymer matrix and selection of suitable conductive filler. Polyurethane (PU) is made up from polyol as soft segment and isocyanate together with 1-6 hexane-diol (chain extender) as hard segment [8]. Both soft and hard segments were separated at microphase due to incompatibility between them. Therefore, by varying segment content, composition and nature of the reagent, different PUs can be tailored. Different soft and hard segments of molecular structures can be generated in these techniques, leading to polyurethane with a variety of properties and applications, including coatings, adhesives, electronic, printing and medicine [8,9,10,11].

Recent increases in global environmental awareness, the mission of lowering organic volatile compounds and climate change guidelines has sparked interest in green materials. Thereby, waterborne polyurethanes (WBPU) have risen to prominence because of their capacity to assemble in stable particles in water dispersions when a covalently bound internal emulsifier is added. Waterborne based PU, which uses water as the solvent or carrier that emits very few volatile organic compounds (VOCs), is recognized as one of the environmentally friendly PU to replace traditional organic solvent-based PU which is widely used in the industry [12,13,14].

Carbon nanomaterials (CNMs) have been widely exploited in a variety of disciplines and their relevance has been reflected in recent nano technological research. Graphene possesses the best qualities of all CNMs, including surface area, light weight, great electrical conductivity, and good thermal and mechanical capabilities [15]. Graphene is the fundamental unit of graphite-like materials, with a two-dimensional single to few atomic layers and a sp2 hybridized structure [16]. Graphene has a significant pricing advantage over other CNMs, such as carbon nanotubes, carbon nanofibers, and activated carbon, due to its wide range of sources [16]. Graphene has been extensively accepted for physical, chemical, analytical, and biological applications [16,17,18,19,20].

Overall, PU and graphene have remarkable advantages as green conductive nanocomposite materials. Two disadvantages remain in this incorporation process. One is that irreversible aggregation is formed because of the strong natural π-π stacking tendency and Van der Waals interaction among the graphene nanoplatelet (GNP). These GNP sheets stacking, and Van der Waals interactions limit their potential applications. This technical hindrance, however, can be addressed by introduce GNP at a very minimum wt.% just slightly above the percolation threshold for effective electrical channeling. Another disadvantage is that the chemical used for processing and dispersion of GNP is hydrazine, hydrazine derivatives, hydroquinone, and sodium borohydride, all of which are highly toxic and damaging to the environment. Considering this, low loading of GNP was incorporated to avoid excessive use of toxic chemical both at production and incorporation stages. Few studies on the use of green incorporation of graphene have been published. For example, Luo et al. [21] incorporated graphene into PU matrix using acetone as solvent medium. The thermal decomposition of the composite was increased by 25 °C by 1 wt.% of GNP. Meanwhile, the conductivity, hydrophobicity, and tensile strength of the material have all been improved. Lee et al. [22] prepared waterborne polyurethane (WBPU) reinforced GNP by an in-situ method. Acetone was used as solvent as well. It is found out that, GNP was finely dispersed as nano size in a WBPU to form an effective conducting channel, thus the conductivity of WBPU was effectively increased with addition of GNP. This demonstrates that the compatibility of GNP with WBPU is sufficient to produce a high-performance nanocomposite without further GNP surface treatment.

The goal was to fabricate jatropha oil based WBPU conductive nanocomposite by introducing very minimum GNP filler that has good conductivity for potential use in conductive coating. To get the best out of WBPU-GNP nanocomposite, 0.5 wt.% cellulose nanofiber (CNF) was added to the system. Unlike GNP, CNF is a hydrophilic in nature that can be blended uniformly with any water-based polymer. Consequently, CNF will provide mechanical support since loading of GNP was at minimal. The work on incorporation of CNF in jatropha oil based WBPU was conducted in our previous studies and 0.5 wt.% shows the optimum loading for CNF which led to 55% and 22% improvement on Young’s modulus and tensile strength respectively [14,23]. Water/moisture behavior must be limited or non-existent due to the importance of polyurethane in the application of the coating. It is expected that, not only conductivity will be enhance, water activity of WBPU-GNP-CNF will be approaching non-exist due to hydrophobic nature of GNP.

As far as our concerned, there is no information or study involving CNF and GNP in waterborne Jatropha oil-based polyurethane. A Jatropha oil-based waterborne polyurethane nanocomposite film containing CNF and GNP as a filler was therefore prepared and characterized in the present study. The effect of CNF and GNP on the physical, mechanical, thermal and conductivity properties of this nanocomposite film will be investigated.

## 2. Materials and Methods

### 2.1. Materials

Formic acid (98%), pyridine (95%), and N-methyl pyrrolidone (NMP) (98%) were purchased from Fisher Scientific, Pittsburgh, PA, USA. Triethylamine (TEA) (30%), hydrogen peroxide, dibutyltin dilaurate (DBTL) (98%), methanol (99.8%), phthalic anhydride, dimethylol propionic acid (DMPA), acetone (reagent grade), sodium hydroxide 0.5 N, and magnesium sulfate was purchased from R&M chemicals, Tamil Nadu, India. Isophrene diisocyanate (IPDI) (98%) and GNP (particle size: <2 µm, surface area: 300 m^2^/g) was purchased from Merck, Darmstadt, Germany, and 1–6 Hexanediol (HDO) was purchased from BDH Chemical LTD, Poole, England. Cellulose nanofiber was obtained from the Institute of Tropical Forestry and Forest Product (INTROP) (Serdang, Malaysia). Crude Jatropha oil was supplied by Biofuel Bionas Sdn Bhd, Kuala Lumpur, Malaysia.

### 2.2. Preparation of Jatropha Oil Based Polyol (JOL)

The method reported by Saalah et al. [24] was used to synthesis epoxidized Jatropha oil (EJO). A four-neck flask with a mechanical stirrer, a torque meter, a heater with a temperature sensor, and a dropping funnel was prepared. The flask was filled with methanol and water, then followed with sulphuric acid. After that, the mixture was heated to 64 °C before adding EJO, and the reaction was allowed to run for 30 min. Next, sodium bicarbonate was added to stop the process. The solution was poured into the separating funnel, allowed to cool to ambient temperature, and then the deposited layer was discarded. Vacuum distillation at 60 °C for 30 min at 100 rpm was used to extract the methanol and water. This produced a clear golden yellow polyol, which was tested for hydroxyl (OH) content.

### 2.3. Preparation of Jatropha Oil Based Polyurethane Dispersion

**Figure 1:**
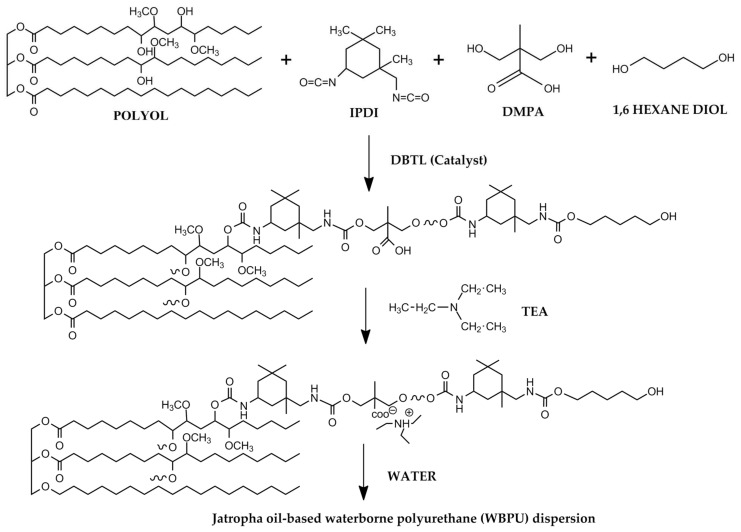
Reaction scheme of WBPU.

A four-neck flask with a mechanical stirrer containing torque meter, a heater with a temperature sensor, nitrogen inlet and a dropping funnel was prepared. The flask was filled with JOL and the calculated amount of DMPA (dissolved in NMP). The mixture was agitated at 400 rpm for 30 min and heated to 70 °C for homogenous mixture. As a catalyst, 1 mL of DBTL was added, and the reaction was set for 30 min. Next, the IPDI was added dropwise over 30 min, and the agitation was raised to 700 rpm. The reaction temperature was increased to 80 °C once the IPDI was completed. At the same time, acetone was added in small batches to control the viscosity of the system. HDO was introduced after another 2 h of reaction time. The reactant was cooled to 35 °C, then TEA was added to neutralise the DMPA, followed by dispersion with deionized water at 1200 rpm to obtain WBPU with a solid concentration of 38 wt.%. Acetone was quickly extracted under vacuum using a rotary evaporator. Table 1 and Figure 1 demonstrate the WBPU’s full formulation and reaction sequence.

### 2.4. Preparation of WBPU-CNF-GNP Nanocomposite Film

**Figure 2:**
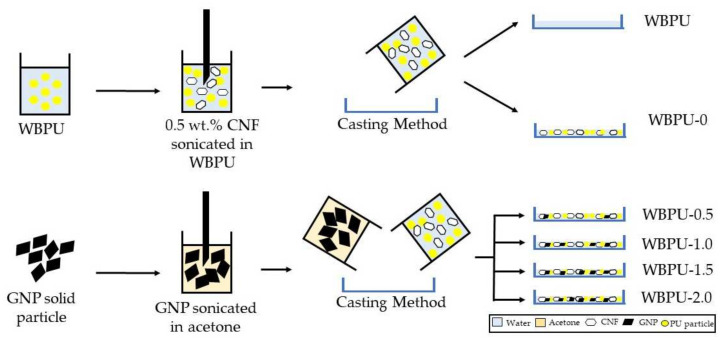
Preparation process of ternary nanocomposite system.

The film casting method was used to make WBPU-CNF-GNP nanocomposites films. 0.5 wt.% CNF was sonicated directly with WBPU solution, while GNP was sonicated with acetone prior addition to WBPU-CNF solution. Each stage of sonication was set to 30 min to obtain well homogeneous mixture. The weight percent of GNP in WBPU was set to 0.5, 1.0, 1.5 and 2.0% respectively. The mixture was then transferred into the Teflon molds and kept for 7 days in the desiccator and 12 h in the vacuum oven at 60 °C. The film was 0.4 mm thick after it was fully cured. According to their GNP loadings, the nanocomposite films were labelled WBPU-0.5, WBPU-1.0, WBPU-1.5 and WBPU-2.0. WBPU reinforced with 0.5 wt.% CNF was fixed for the entire studies and named as WBPU-0. Neat WBPU and WBPU-0 was prepared as references. Details preparation of ternary nanocomposite system is illustration as shows in Figure 2.

### 2.5. Characterisation

#### 2.5.1. Fourier Transform Infrared Spectroscopy (FTIR) Characterisation

The chemical structure of the nanocomposite films was analyzed using FTIR spectra by Perkin-Elmer, Spectrum 2000 series, manufactured in the Beaconsfield, United Kingdom equipped with horizontal germanium attenuated total reflectance (ATR). The spectra were collected with a nominal resolution of 4 cm^−1^, in the range of 4000 to 500 cm^−1^.

#### 2.5.2. Mechanical Properties Characterisation

The mechanical properties of the nanocomposite films were determined by the INSTRON, 3300 series universal tensile machine, made in the Norwood, MA, USA, as specified by ASTM D638-03 Type V. The crosshead speed was 10 mm/min, with a load cell of 1 KN. Young’s modulus, tensile strength, and elongation at break were calculated using data from stress and strain measurements. Each sample’s value was calculated using an average of three measurements. The test was conducted at room temperature with a relative humidity of 50%.

#### 2.5.3. Thermal Degradation Analysis

The thermal property of the nanocomposite films was analysed using a TA Q500 series analyzer produced in New Castle, DE, USA. The films were heated from 25 to 600 °C, at a rate of 10 °C/min, under nitrogen environment.

#### 2.5.4. Morphology Evaluation

The morphology of the nanocomposite films was examined using a field emission scanning electron microscope (FESEM) by Thermo Fisher Scientific, Nova Nanosem 230 series, manufactured in the MA, USA. A layer of gold was sputtered onto the samples. The images were then magnified up to 50 K magnification with a 5 kV accelerating voltage.

#### 2.5.5. Water Uptake Analysis

Water uptake measurement is one of the most essential characteristics of hydrophilic composite films, especially since CNF is a hydrophilic water-like substance while GNP is hydrophobic. The water uptake was determined using the method proposed by Fang et al., 2014 [25]. All nanocomposite films were sliced into 5 mm with triplicate samples. The sliced samples were submerged in deionized water for 5 days. The weight of the specimens was taken after 2, 6, and 12 h, and then every 24 h after that. The difference in sample weight was used to calculate the percentage of water uptake of each specimen, as shown in Equation (1)

(1) $$\mathrm{Water}\;\mathrm{uptake}\;\left(\%\right)=\left[(W_{b}-W_{a})/W_{a}\right]\times 100$$

where W_a_ is the initial weight of the specimen before immersion and W_b_ is the weight after immersion, respectively.

#### 2.5.6. Water Contact Angle

Because CNF is a hydrophilic water-like material and GNP is hydrophobic, the film’s response to water absorption was observed using the water contact angle test. The static contact angle was observed at room temperature on the surface of the all-nanocomposite films using the Attension Theta Optical Contact Angle Tester (Biolin Scientific, Manchester, UK). The 3 µL of deionised water were dropped onto the film surface using the sessile dropping method. Within one minute after the water had dropped, the contact angle data were recorded based on surface interaction

#### 2.5.7. Conductivity Analysis

The conductivity of pristine WBPU, WBPU-0 and WBPU reinforced by GNP was determined using two-point probe by Keithley Instruments, Keithley 2400 source meter, manufacture in the Ohio, USA. Resistivity measurement for WBPU, WBPU-0 and WBPU reinforced GNP was performed on round shape sample with 2 cm radius held by sample cell. Analysis was repeated at least three times and converted into conductivity using the thickness of the sample.

## 3. Result and Discussion

### 3.1. Fourier Transform Infrared Spectroscopy (FTIR)

**Figure 3:**
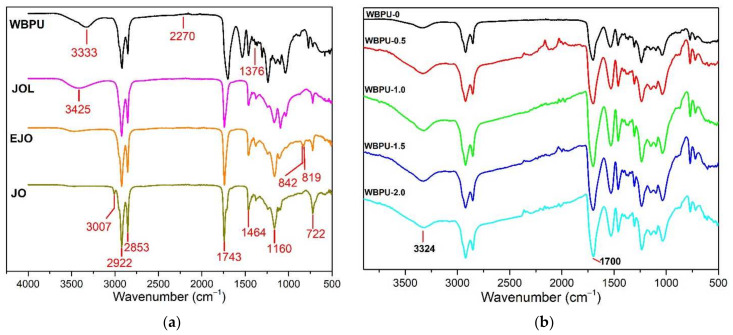
(**a**) FTIR spectra of derivation of WBPU from jatropha oil; (**b**) FTIR spectra of WBPU, WBPU-0, WBPU-0.5, WBPU-1.0, WBPU-1.5 and WBPU-2.0.

Figure 3a shows the results of FTIR analysis for production of jatropha oil based waterborne polyurethane. The explanation of FTIR spectra on the derivation of jatropha oil based WBPU from jatropha oil and incorporation of different loading of CNF onto WBPU was discussed in detail on our previous studies [14,23].

Figure 3b shows the FTIR spectra plots of the different GNP loadings for the WBPU nanocomposite. All WBPU and WBPU-0 nanocomposite characteristic bands have been detected. The peaks located between 3380 and 3445 cm^−1^, and 1700 cm^−1^ corresponding to stretching vibration of NH and OH groups (overlapped peaks) and hydrogen bonded carbonyl group bonding, respectively, appeared and grew over time. As loading of GNP increases from 0.5 to 2.0 wt.%, the peaks of NH and OH groups (overlapped peak) were shifted to higher wave numbers while hydrogen bonded carbonyl group was increase in peak intensities, respectively. This observable peak is a result of hydroxyl and carbonyl group detected on the edge of GNP. The results obtained proved the presence of GNP in WBPU-CNF-GNP nanocomposite system. Interfacial interaction would only be allowed if there is any crosslinker used and surface modification made on GNP or CNF [25,26]. Furthermore, it is hard to discover specific interaction between GNP and CNF either by covalent or non-covalent bond with FTIR analysis alone. The details of all FTIR characteristic band are tabulated in Table 2.

### 3.2. Surface Morphology

**Figure 4:**
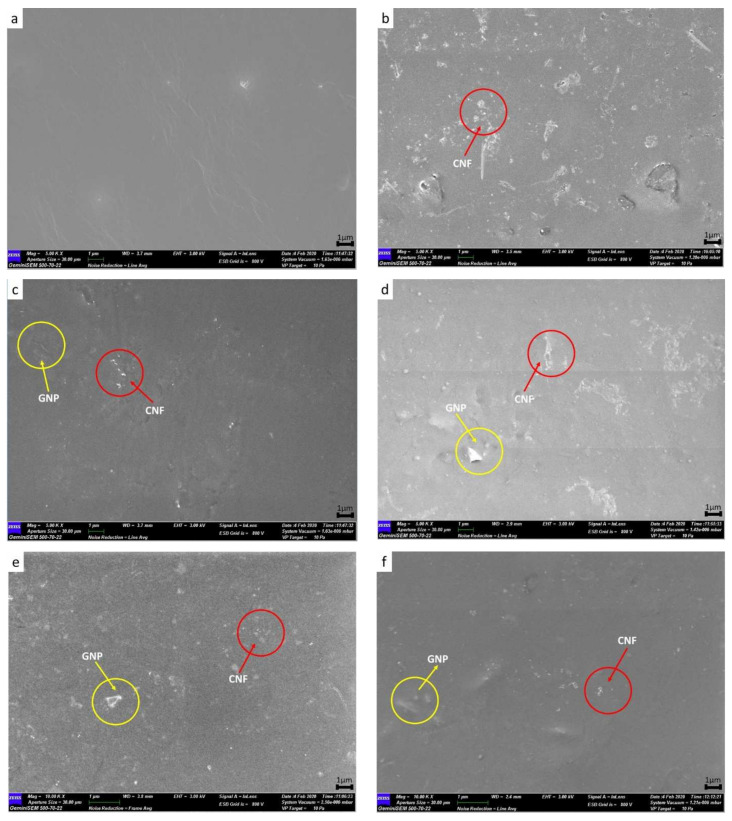
FESEM image of: (**a**) WBPU; (**b**) WBPU-0; (**c**) WBPU-0.5; (**d**) WBPU-1.0; (**e**) WBPU-1.5; (**f**) WBPU-2.0.

The FESEM morphology of the WBPU and WBPU-0 to WBPU-2.0 nanocomposite films is depicted in Figure 4. The surface of WBPU shows smooth, neat, and clean (Figure 4a). As compared to WBPU-0, surface grew rougher with addition of 0.5 wt.% CNF (Figure 4b).

CNF nanoparticle may be seen as white dots on the FESEM images of WBPU-0 as indicated by red circle. The WBPU-0 displays a good distribution of CNF in the WBPU matrix which is prove by the white dots with nano size equally placed around WBPU matrix. In the meantime, it revealed the compatibility and existence of interactions between hard segment of WBPU and CNF. FESEM image with similar characteristics have been described in others literature [27,28,29,30]. It should be mentioned at this point that 0.5 wt.% is the optimum CNF loading for incorporation onto jatropha oil based WBPU as addressed on our previous studies [14,23]. This is important that, the optimum loading and homogenous distribution of the CNF in the matrix play an important role in improving the mechanical properties of the resulting nanocomposite films.

**Figure 5:**
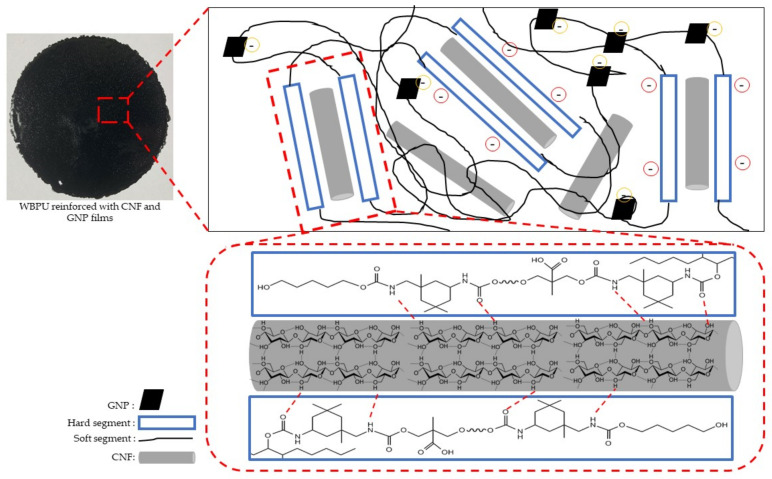
The schematic diagram of electrostatic force and distribution of CNF and GNP.

GNP was chosen as conductive filler to enhance electrical properties of non-conductive WBPU-0 nanocomposite system. Therefore, effect incorporation of GNP on morphology of WBPU was investigated. Figure 4c–f show morphology images of the nanocomposite system comprising of WBPU, CNF and GNP. GNP was observed on the surface as an extrude nanoparticle, as indicated by the yellow circle. By comparing surface of WBPU-0 (Figure 4b) and WBPU-0.5 to WBPU-2.0 (Figure 4c–f) nanocomposite series, white dots and extruded nanoparticle was observed to be distributed evenly throughout WBPU-CNF-GNP surface morphology. In fact, GNP was easily distributed, despite presence of CNF as no large aggregated extruded nanoparticle was observed. GNP was seen as extruded nanoparticle was due to electrostatic repulsion of negatively charge of GNP. Under neutral condition the residual carboxyl group may dissociate to –COO-group, which imparts the GNP surfaces to a negative charge. Furthermore, negative charge GNPs are incompatible with the WBPU matrix, which contains negative hydrophilic functional groups because of the TEA neutralization process. Present of carboxyl group on surfaces of GNP was supported by FTIR. In addition, the negative charge on GNP surfaces is so weak due to the minimal amount of carboxyl groups, therefore self-exfoliation to a single layer is not efficient [31,32]. This trend was also in agreement with findings of other researchers [21,33,34,35]. The schematic diagram of electrostatic repulsion force and distribution of CNF and GNP is presented in Figure 5.

### 3.3. Mechanical Properties

**Figure 6:**
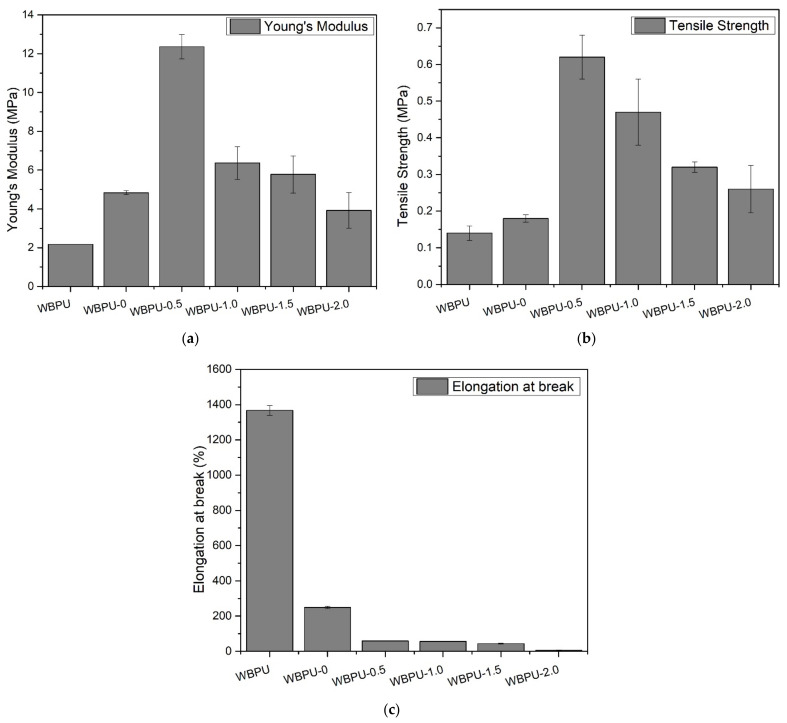
Mechanical properties of nanocomposite films: (**a**) Young’s modulus; (**b**) Tensile strength and (**c**) Elongation at break.

The tensile strength, Young’s modulus and elongation at break plot of the mechanical properties for nanocomposite films are presented in Figure 6. As expected, the Young’s modulus and tensile strength value increased while elongation at break decreased with addition of 0.5 wt.% CNF. Young modulus and tensile strength were enhanced up to ~122% and ~28%, respectively. This is because of 0.5 wt.% is a very low loading which showed no excess loading of the filler as it is below the percolation threshold. CNF provides interconnected cellulosic network as a mechanical support within polymer. The network formed was assisted by the flexibility of the CNF due to the existence of amorphous domains along the nanofibers and its high aspect ratio [29]. The findings are in line with PU composites reinforced with conventional fillers [36,37,38,39,40,41].

The Young’s modulus and tensile strength of WBPU-0.5 to WBPU-2.0 increase with increasing GNP content until it reaches a maximum value at 0.5 wt.% and thereafter decrease as loading of GNP increase from 1.0 wt.% to 2.0 wt.%. This is believed to occur due to the present of negatively charge ions located on surface of GNP and hard segment chain of polymer matrix. Electrostatic repulsion force exerted between GNP and polymer matrix which led GNP approaching agglomeration [42]. It is known that graphitic material does not compatible with water. Since WBPU comprising of water and PU particle, GNP still faces a problem to be well dispersed even though it was dispersed in acetone prior addition into WBPU solution. Long duration of time for curing process provides enough timeframe for GNP to restack [22,33].

The elongation at break decreased as GNP loading increased while CNF was constant at 0.5 wt.%. When polyurethane stretched, the hard segment domains disintegrated, resulting in some phase mixing between the soft and hard segments, and both segments orientated toward the elongation, leading in the greatest intermolecular contact. When the intermolecular interaction is at its peak, it prevents the molecular rearrangement of WBPU, CNF and GNP. Since there are 2 fillers used, both CNF and GNP forming a highly compact structure, thus, decreased the physical interaction area, resulting in a stress deficiency transfer [43]. This area of agglomeration and heterogeneous has become a stress concentrator and a failure point for nanomaterial composites film.

Overall, the distinctive properties of CNF, as mechanical reinforcement, were not affected by the presence of conductive filler, as both Young’s and tensile strength was enhanced and no decreased in mechanical was observed below value set by WBPU-0. Therefore, it is worth mentioning that 0.5 wt.% of CNF and 0.5 wt.% of GNP was the optimum loading needed to enhance mechanical properties of this ternary nanocomposite system.

### 3.4. Thermal Properties

**Figure 7:**
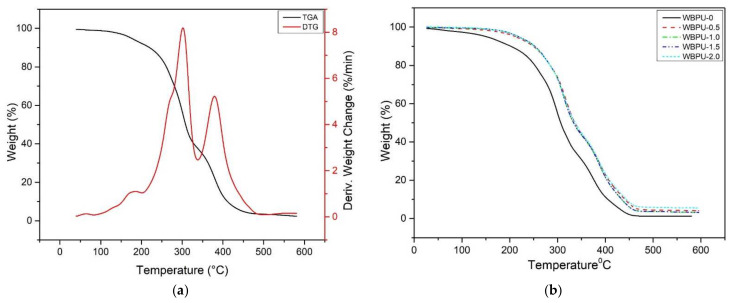

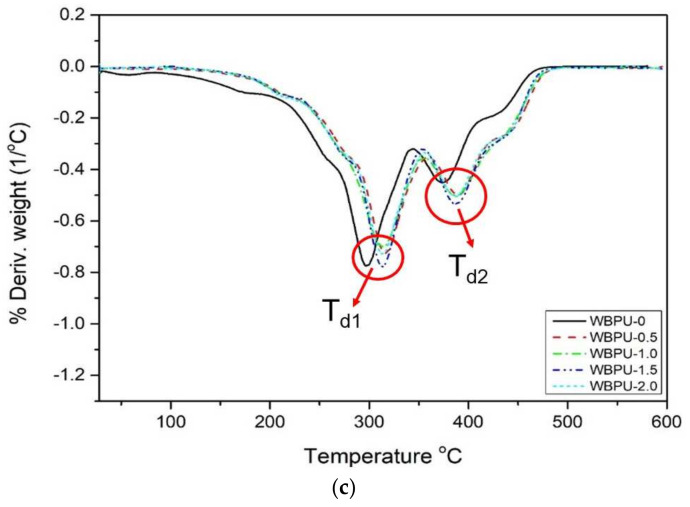
Thermal properties of: (**a**) TG and DTG curve for WBPU; (**b**) TGA and (**c**) DTG curve of WBPU-0, WBPU-0.5, WBPU 1.0, WBPU-1.5 AND WBPU-2.0.

Thermogravimetric analysis was used to investigate the thermal degradation of nanomaterials. Figure 7a–c show the TG and DTG thermograms of all nanocomposite films, while Table 3 summaries all the thermal parameters. Details on thermal degradation of WBPU were discussed thoroughly in our previous studied [14,23]. In brief, neat WBPU experienced a two-step weight loss detected at ~299 °C and ~378 °C, respectively. The thermal degradation of the hard segment (urethane group) and soft segment (JOL component) in the WBPU chains resulted in weight losses of 53.82% and 40.72%, at T_d1_ 299 °C and T_d2_ 378 °C, respectively.

In case of WBPU-0, as 0.5 wt.% of CNF added, the first and second temperatures of thermal degradation, T_d1_ and T_d2_, of the nanocomposite film shifted to higher temperatures, as indicated in Figure 6b,c. The mechanical interaction between WBPU and CNF, as explained in the FTIR analyses mentioned above, was believed to be responsible for the improved thermal stability of the nanocomposite film. Meanwhile, it clearly shows that, thermal degradation of the nanocomposite system was greatly affected by GNP loading. The thermal degradation temperature for WBPU-0.5 to WBPU-2.0 increased with increasing GNP content until 0.5 wt.% and then plateau for 1 wt.% to 2 wt.%. This is thought to be due to 2 factors; (1) the graphene properties itself as their initial degradation temperature is over 2800 °C under vacuum condition and (2) physical interaction with polymer matrix and CNF. CNF which believed has specific mechanical interaction with hard segment, decomposed at a lower temperature (T_d1_) to form a char layer on the nanocomposite film, acting as a retardant or barrier to the decomposition of the soft segment at a high temperature [44]. Additionally, GNP, a sheet like material with high aspect ratio and little weight loss up to 700 °C, act as second barrier after CNF, hindered the diffusion of the volatile decomposition product and further enhance the char formation, thus provide thermal support for the nanocomposite system [36]. Apart from that, the chain transfer reaction was delayed by graphene during the thermal degradation process [45]. Due to the electrostatic repulsion, soft segment area of polymer matrix was covered by GNP. This is proven by enhancement up to 40% of less weight loss of soft segment as compared to pristine WBPU. The same thermogram pattern were also reported in other literatures [37,44,45,46,47].

### 3.5. Water Contact Angle Measurement

**Figure 8:**
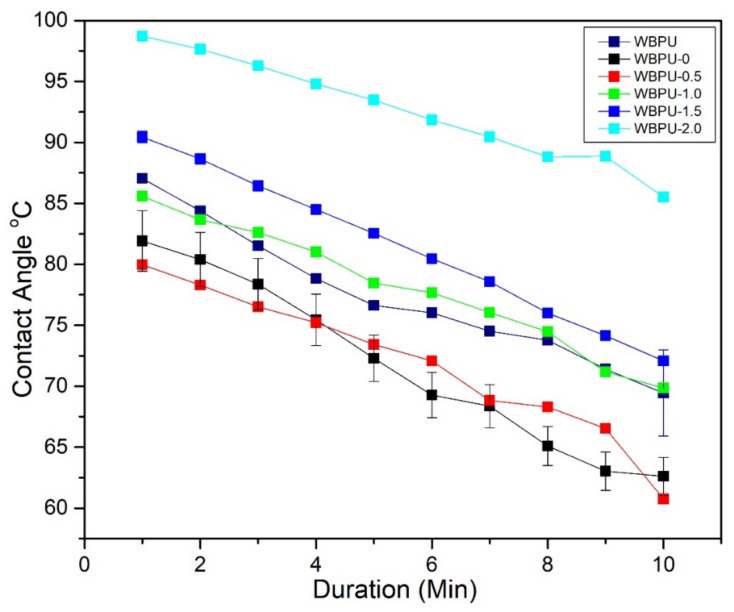
Contact angle of WBPU, WBPU-0, WBPU-0.5, WBPU-1.0, WBPU-1.5 and WBPU-2.0.

The hydrophobicity/hydrophilicity of the film surfaces of all nanocomposite films was observed by measuring the static contact angle, as shown in Figure 8. During the test, the water droplets hardly changed on the pristine WBPU film’s surface, suggesting that the film has only absorbed a small amount of water. This was due to nature of PU properties which is hydrophobic [48]. When 0.5 wt.% CNF was incorporated, the contact angle decreased gradually. Two reasons can be associated to this cause: (1) the hydrophilic/hydrophobic nature of the nanofiller [49] and (2) high surface roughness and lower surface free energy [50]. This is supported by FESEM images which show WBPU-0 has a little of surface roughness as compared to WBPU. On the other hand, the contact angle gradually increased as the GNP content increased from 0.5 to 2 wt.% despite the presence of hydrophilic CNF. This is due to the hydrophobic property of GNP, which causes an increase in contact angle [51].

Surface roughness of nanocomposite films reinforced with GNP is better (less roughness) as compared to WBPU-0. CNF helped increase the alignment degree of GNP sheets which lead to better GNP distribution with more compact morphology and fewer void spaces. More compact morphology and fewer void spaces reduced water transport through nanocomposite films [52]. Overall, the contact angles of WBPU and WBPU-0 nanocomposite films are in between 79° and 87°, which were approaching hydrophobic (90°). However, with addition of 2 wt.% GNP, the nanocomposite film was totally shifted to hydrophobic and neglect the hydrophilic properties of CNF.

### 3.6. Water Uptake Measurement

**Figure 9:**
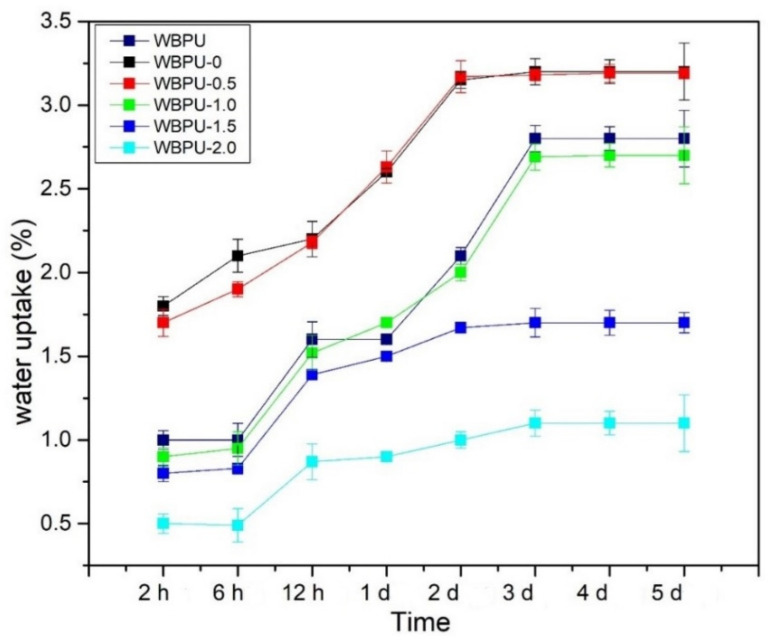
Water Uptake of WBPU, WBPU-0, WBPU-0.5, WBPU-1.0, WBPU-1.5 and WBPU-2.0.

The water uptake as a function of time for all nanocomposite films is shown in Figure 9. The water uptake for all nanocomposite films increased with time and plateaued at three days and above. In case of WBPU, the water uptake is considerable low which in range of 1–3% over 10 days of immersion. This was due to natural hydrophobic properties of the PU which consist of high hard segment content that was responsible for the high cross-linking density of hydrogen bonding between the segmental PU [53]. The water uptakes of WBPU-2.0 and WBPU-1.5 were the first and second lowest, respectively. Meanwhile, WBPU-0 and WBPU-0.5 were the highest. Lowest water uptakes by WBPU-2.0 and WBPU-1.5. Both films have better GNP distribution as proven by FESEM images. Addition of GNP helps further enhanced the hydrophobicity properties of WBPU-2.0 and as GNP is hydrophobic material. Therefore, better GNP distribution provides tortuous path and increases the barrier property for water transport [54]. Another reason for less water absorption could be the water repelling nature of GNP that tends to immobilize some of the moisture, which inhibits the water permeation in the polymer matrix. The highest water uptakes by WBPU-0 and WBPU-0.5 were mainly attributed by hydrophilic nature of CNF. Even with addition of 0.5 wt.% GNP, it is not enough to cover overall PU films, therefore, hydrophilic properties of CNF are dominant. All data related to water absorption were led by hydrophilic cellulosic material in the composite as well as void spaces and micro gaps at the interface. It can be concluded that water uptakes of WBPU, WBPU-0 nanocomposite films were low and did not exceed 3.5%. With addition of GNP, the hydrophobicity was further enhanced. This was supported by the result obtained from the contact angle of 90° to 100°, which indicates that those films are totally hydrophobic.

### 3.7. Conductivity Properties

**Figure 10:**
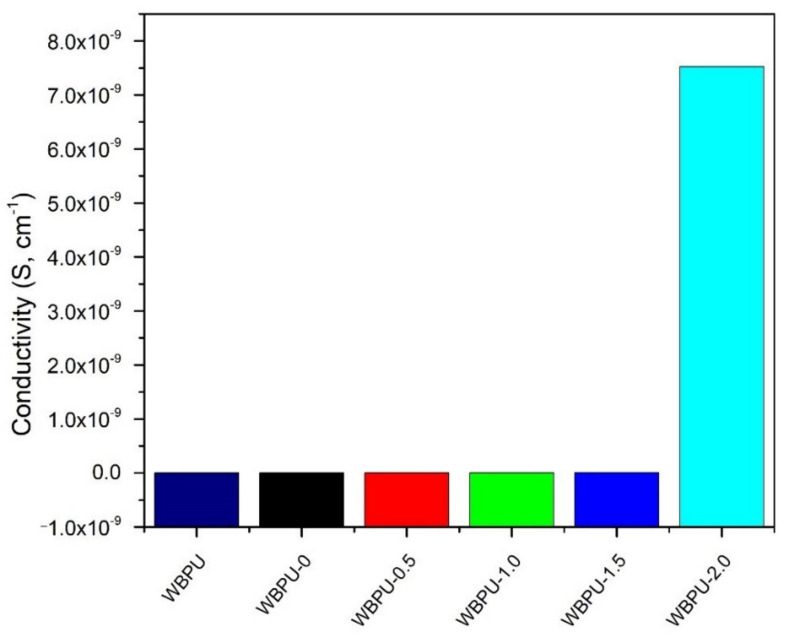
Conductivity profile of all nanocomposite films.

Figure 10 shows the conductivity profile of all nanocomposite films. The conductivity was gradually increased as the GNP content increased from 0.5 to 2 wt.%.

The increase in conductivity with increasing GNP content can be explained by finely dispersed GNP that makes an effective conducting network even at very low content, as shown in FESEM images. However, result obtained here does not achieved desirable conductivity values as reported by other researchers. Cai & Song [55] prepared the WBPU reinforced with carbon-based filler treated by surfactant, whose conductivity was about 10^−8^ S.cm^−1^ at 1 wt.% loading. As compared to present study, surfactant used by Cai & Song [55] helped carbon-based filler to have better dispersion with WBPU matrix as GNP is hydrophobic. This led to GNP can be precipitated easily during dispersion and curing processes of nanocomposite films. However, these results show that the GNP can be utilized for the improvement of the conductivity of WBPU. Further improvement can be made if appropriate surfactant is used for better GNP dispersibility.

## 4. Conclusions

In this work, Jatropha oil-based waterborne polyurethane nanocomposite reinforced with CNF and GNP was successfully prepared, characterised, and tested. We determined the effect of GNP filler with fixed CNF loading (0.5 wt.%) on mechanical, thermal, water uptake behavior, contact angle and conductivity of the nanocomposite film. CNF and GNP was successfully incorporated in WBPU matrix as proved by FTIR spectra. GNP was easily distributed evenly throughout WBPU-CNF-GNP surface morphology despite the presence of CNF as no large aggregated extruded nanoparticle was observed on FESEM images. An optimum effect on mechanical properties was observed on WBPU-0.5. This could be attributed to the less electrostatic repulsion force as low GNP loading was incorporated, thus, nanocomposite with 0.5 wt.% GNP shows better homogeneity reaffirmed by FESEM images. Thermal degradation temperature shifted to higher temperature as loading of GNP increase. This is thought to be due to 2 factors which are high initial degradation temperature of graphene and physical interaction with polymer matrix and CNF.

The contact angles of WBPU-0.5 to WBPU-2.0 nanocomposite films are increased due to more compact morphology and fewer void space which reduce water transport of nanocomposite film. With 2 wt.% GNP, nanocomposite film was totally shifted to hydrophobic and neglect the hydrophilic properties of CNF. Furthermore, the water absorption activities films of WBPU-0.5 to WBPU-2.0 is the lowest because of better GNP distribution provides tortuous path which increase the barrier property for water transport and immobilize some of the moisture, inhibits the water permeation in the polymer matrix. Overall, with addition of GNP, the contact angle obtained is between 90° to 100°, which indicate totally hydrophobic. Lastly, the desirable conductivity was not achieved due to GNP precipitated easily during dispersion and curing processes of the films.

## Figures and Tables

**Table 1:** Formulation of WBPU synthesis.

	GNP ^a^	CNF ^a^	JOL ^a^	DMPA ^a^	IPDI ^a^	HDO ^a^	TEA ^a^	Water ^a^
WBPU	0	0	52.54	11.2	24.52	5.17	6.57	16
WBPU-0	0	0.5	52.54	11.2	24.52	5.17	6.57	16
WBPU-0.5	0.5	0.5	52.54	11.2	24.52	5.17	6.57	16
WBPU-1.0	1	0.5	52.54	11.2	24.52	5.17	6.57	16
WBPU-1.5	1.5	0.5	52.54	11.2	24.52	5.17	6.57	16
WBPU-2.0	2	0.5	52.54	11.2	24.52	5.17	6.57	16

^a^ Unit is in weight percentage.

**Table 2:** FTIR characteristic band of WBPU, CNF and GNP.

Band Assignment	Wavenumber (cm^−1^)
Neat WBPU	
${ʋ}_{N-H}$	~3600–3000
${ʋ}_{C=O,H-bond\;urethane,}$	~1700
${ʋ}_{N-H}/\delta_{N-H}$	~1550
$\delta_{C-H,\;sym}$	~1376
CNF	
$\nu_{O-H}$	~3372
$\nu_{C-H}$	~2894
$\nu_{C-O}$	~1592
$\nu_{C-O-C}$	~1059
GNP	
$\nu_{O-H}$	~3324
${ʋ}_{C=O,}$	~1700

**Table 3:** Thermal properties of nanocomposite films.

	T_dec_ (°C)		Weight Loss (%)		Residue at 600 °C (%)
	T_d1_	T_d2_	W_d1_	W_d2_	
WBPU	299.76	378.79	53.82	40.72	0.9962
WBPU-0	313.42	380.25	60.70	35.25	0.0001
WBPU-0.5	316.28	389.25	53.43	25.98	0.0001
WBPU-1.0	315.18	388.19	53.55	24.66	0.0001
WBPU-1.5	316.55	388.30	51.59	28.87	0.0001
WBPU-2.0	316.05	387.88	53.84	24.37	0.0001

## Data Availability

The data presented in this study are available upon request from the corresponding author.
